# Modeling and Characterization of Complex Concentrated Alloys with Reduced Content of Critical Raw Materials

**DOI:** 10.3390/ma14185263

**Published:** 2021-09-13

**Authors:** Beatrice-Adriana Șerban, Ioana-Cristina Badea, Nicolae Constantin, Dumitru Mitrică, Mihai Tudor Olaru, Marian Burada, Ioana Anasiei, Simona-Elena Bejan, Andreea-Nicoleta Ghiță, Ana Maria-Julieta Popescu

**Affiliations:** 1 National R&D Institute for Non-Ferrous and Rare Metals, 102 Biruinței, 077145 Pantelimon, Romania; beatrice.carlan@imnr.ro (B.-A.Ș.); dmitrica@imnr.ro (D.M.); o.mihai@imnr.ro (M.T.O.); mburada@imnr.ro (M.B.); sbejan@imnr.ro (S.-E.B.); andreea.lupu9@yahoo.com (A.-N.G.); 2 Engineering and Management of Obtaining Metallic Materials Department, University Politehnica of Bucharest, 313 Splaiul Independentei, 060042 Bucharest, Romania; nctin2014@yahoo.com; 3 Romanian Academy, “Ilie Murgulescu” Institute of Physical Chemistry, 202 Splaiul Independenței, 060021 Bucharest, Romania; popescuamj@yahoo.com

**Keywords:** complex concentrated alloys (CCAs), simulation, critical raw materials (CRMs), elaboration, properties

## Abstract

The continuous development of society has increased the demand for critical raw materials (CRMs) by using them in different industrial applications. Since 2010, the European Commission has compiled a list of CRMs and potential consumption scenarios with significant economic and environmental impacts. Various efforts were made to reduce or replace the CRM content used in the obtaining process of high-performance materials. Complex concentrated alloys (CCAs) are an innovative solution due to their multitude of attractive characteristics, which make them suitable to be used in a wide range of industrial applications. In order to demonstrate their efficiency in use, materials should have improved recyclability, good mechanical or biocompatible properties, and/or oxidation resistance, according to their destination. In order to predict the formation of solid solutions in CCAs and provide the optimal compositions, thermodynamic and kinetic simulations were performed. The selected compositions were formed in an induction furnace and then structurally characterized with different techniques. The empirical results indicate that the obtained CCAs are suitable to be used in advanced applications, providing original contributions, both in terms of scientific and technological fields, which can open new perspectives for the selection, design, and development of new materials with reduced CRM contents.

## 1. Introduction

Metallic materials have extended their application spectrum with protometallurgy, when meteorite iron was used for the manufacturing of weapons, household items, or tools. Later, human communities learned to exploit and process the underground ores and people were able to see the benefits that these activities bring. To simplify the quotidian activities and increase productivity, people began to alloy the subterranean materials discovered and developed metallurgy—an important contribution for humankind [1,2].

Precious metal alloys have had great importance in medical applications, with gold being used in dental applications for over 2000 years. However, for reasons of ancient religious dogmas, the medical applications were slowed down until 1500 A.D. Despite all these impediments, in 659 B.C., a metallic material with applicability in dental medicine was developed, containing 100 parts of mercury, 45 parts of silver, and 900 parts of tin [3].

Up to the present, the most used metallic materials for the manufacturing of medical implants are Co–Cr–Mo and Ti alloys. They have superior mechanical and biocompatible properties, so they can be used for the replacement of artificial joints (hip, knee, and shoulder prostheses). Although they have good biocompatible characteristics, titanium alloys (e.g., Ti_6_Al_4_V) cannot be used as contact surfaces between two materials due to the low wear resistance, which is associated with low shear strength and repassivation behavior of the oxide layer from the surface [4]. Alloys from the Co–Cr–Mo system (e.g., Co_28_Cr_6_Mo) have higher wear resistance and can be used as contact surfaces for joint prostheses, but there are identified situations where the implant has been rejected by the human body [5]. It has been observed that the level of chromium and cobalt metal ion increases in the blood of patients with implants from the analyzed alloys’ systems. In vivo and in vitro tests were also performed to demonstrate the risk of cytotoxicity, DNA damage, hypersensitivity reactions to metal and pseudo-tumors [6]. The materials currently used in the manufacturing of implants have many disadvantages, so the development of new materials that are resistant to wear and prolong the life of implants remains a topical challenge [7].

Civilizational progress has led to the development of new materials with improved properties, based on exhaustible mineral resources. This has favored an irrational consumption of the existing natural resources, which can compromise the ability of future generations to meet their needs [8]. To prevent this and to promote the sustainable development-exploiting tendency of mining resources, the European Union has compiled a report containing the global situation of critical metals and materials [9]. This class of materials is widely used in high-tech products, and industrialized societies are dependent on them [10]. This study included the potential consumption scenarios and the necessity to move to a climate-neutral economy through the development of new technologies that decrease the critical material demand and the industrial ecological footprint. Table 1 presents the critical raw materials list, which was first published in 2020.

To reduce or totally replace the critical metals from the composition of advanced materials some changes have been made, but challenges arise when the performances of the obtained product depended on them. For example, silicon is necessary for semiconductors, titanium is a key element in biocompatible materials, and magnesium provides strength and corrosion resistance in light alloys [9].

To decrease the high demand for critical materials, various strategies have been developed to modify the established technologies or to design new materials with similar properties but with a lower content of CRMs [10].

Commonly, metallic alloys are based on the existence of a major element that influences the properties of the material with the addition of alloying elements, which improve the final structure of the metallic material [11]. Complex concentrated alloys (CCAs) [12] are a new family of materials that have a distinct design strategy, due to their multitude of attractive characteristics, such as high hardness, good wear resistance, fatigue resistance, superior thermal properties, low elasticity modulus values, and/or increased oxidation and corrosion resistance [13]. Complex concentrated complex alloys are extending the boundaries of high entropy alloys by accepting a smaller number of alloying elements, which can influence the formation of mixed structures based on solid solutions and intermetallic phases. CCAs have a higher configurational entropy value, have no single, dominant element, and can form structures based on disordered solid solutions [14,15].

The multi-principle nature of complex concentrated alloys and the unique structures they produce have a significant effect on reducing the critical raw material contents in biomaterials with specific properties [16]. The structural particularities of CCAs increase the recyclability rate, which is relevant for the current societal concerns regarding environmental protection and the need to reduce the industrial ecological footprint [17].

To better predict the structure and to develop the most appropriate compositions of CCAs, thermodynamic and kinetic criteria were calculated. In order to have a more complex selection process for CCA compositions, the CALPHAD modeling method was used, as well as for thermodynamic evaluations and empirical data [18]. The kinetic modules included in the simulation program were based on the solid-state phase transformations, especially considering the accuracy of the calculations performed and their applicability to multicomponent systems [19,20,21].

The focus of this work was on developing new complex concentrated alloys with advanced properties, such as increased oxidation resistance, low density, good surface and mechanical properties, and/or biocompatibility [22]. The constituent elements of the alloy system are already used in conventional alloys, but the main purpose was to reduce or totally replace, where it is possible, the critical raw material contents from CCA compositions, without having a negative influence on the material properties. Another important aspect was to design an inexpensive alloy that can be obtained using common manufacturing processes.

## 2. Materials and Methods

To obtain the required mechanical and physical characteristics, it was very important to identify an appropriate composition of the complex concentrated alloys. The constitutive elements have a strong influence on CCA properties, but it is important to meet the needs of the present society by reducing the content of the used critical raw materials. To reduce the alloy density, there can be used chemical elements such as Ti, Al, Mg, or Si. Good anti-corrosive and mechanical properties or improved tensile strength can be obtained by adding Ti and Nb; mixing them with Zr, the alloys were able to form ternary solid solutions and had improved thermal properties and superplasticity. Mn presence favored the formation of solid solutions structures and Fe improved wear resistance and machinability. Therefore, by introducing inexpensive elements to the alloy, the final cost of the alloy was reduced, which has a favorable economic impact.

The most suitable CCA compositions were selected using semi-empirical criteria [23], which were defined by the following equations:

- Using Boltzmann’s equations, the mixing entropy was determined:
  (1) $${\Delta S}_{\mathrm{mix}}=-R\cdot \sum c_{i}\cdot {\mathrm{lnc}}_{i},$$
  where R is the gas’s constant (8.314 J/molK); c_i_ is the molar fraction of the element i.
- The enthalpy of mixing (ΔH_mix_) was calculated using the Miedema macroscopic model [24]:
  (2) $$∆H_{\mathrm{mix}}=\sum 4c_{i}c_{j}\cdot ∆H_{\mathrm{ij}},$$
  where ΔH_ij_ is the binary enthalpy of the alloy; i and j are the elements introduced to the mix.
- The atomic size difference (δ) was calculated using Equation (3) [15]
  (3) $$\delta =100\cdot \sqrt{\sum c_{i}\cdot {\left(1-\frac{r_{i}}{\;\bar{r}}\right)}^{2}},$$
  where r_i_ is the atomic radius of element i; $\;\bar{r}$ is the average atomic radius.
- The derived parameter Ω, which is influenced by the mixing entropy and the mixing enthalpy [25], was calculated using the following equation:
  (4) $$\Omega =T_{m}\Delta S_{mix}/\left|\Delta H_{mix}\right|\;,$$
  where *T_m_* is the melting temperature calculated with $T_{m}=\sum c_{i}\times T_{mi}$, where *T_mi_* is the melting temperature of element i.
- The Allen electronegativity difference (Δχ) was calculated using Equation (5) [26]:
  (5) $$∆\chi^{\mathrm{Allen}}\;=100\cdot \sqrt{\sum c_{i}\cdot {\left(1-\frac{\chi_{i}}{\;\bar{\chi }}\right)}^{2}},$$
  where $\chi_{i}$ is the electronegativity after Pauling for element i; $\;\bar{\chi }$ is the electronegativity average.
- The valence electron concentration (VEC) was calculated (Equation (6)) to determine the type of solid solution that is found in the alloy [27]:
  (6) $$VEC=\sum c_{i}\cdot {VEC}_{i},$$
  where *VEC_i_* is the valence electron concentration of element i.
- The geometrical parameter (Λ) depends on the mixing entropy (ΔS_mix_) and the atomic size difference (δ) of the mixture [28], expressed as follows:
  (7) $$\Lambda ={\Delta S}_{\mathrm{mix}}/\delta^{2},$$

The thermodynamics and kinetics of the systems were simulated with the MatCalc Pro edition software, version 6.02. The thermodynamic modeling was based on the CALPHAD method (CALculation of PHAse Diagrams) analysis and the kinetic modules were developed based on solid-state phase transformations, taking into account the applicability of these models for multicomponent systems.

The selected alloys were prepared by mixing the technically pure raw materials of Fe, Ta, Nb, Ti, Zr, and Mn to obtain a charge of 350 g for each alloy composition. The alloys were placed in an alumina–zirconia crucible and melted in an induction furnace—Linn MFG, 300 type—under an inert atmosphere. The heating rate was related to the induction coupling capacity and the power applied. In this case, a 20% coupling at 22 kW of power and a 14 kHz frequency allowed for a heating rate of 75 °C/min. The alloy melt was cast inside the furnace in a cylindrical copper mold and then cooled in the furnace under vacuum.

To obtain more homogeneity in the alloy, certain alloys were remelted in the same working conditions and the obtained samples were characterized by structural, physical, and chemical analyses.

The resulting alloys were chemically analyzed by inductively coupled plasma spectrometry (ICP-OES) using an Agilent 725 spectrometer (Agilent, Santa Clara, CA, USA) to determine the percentage of chemical elements. Optical microscopy was accomplished using a Zeiss Axio Scope A1m Imager microscope (Zeiss, Jena, Germany), with bright field, dark field, DIC, and polarization characteristics, and high-contrast ECEpiplan 109/509/1009 lenses. SEM-EDAX characterization was performed with a high-resolution scanning electron microscope, FEI Quanta 3D FEG (FEI Europe B.V., Eindhoven, Netherlands), which was equipped with an X-ray spectrometer (EDS). The configuration of the phases was inspected using an X-ray diffractometer, type BRUKER D8 ADVANCE (Bruker Corporation, Billerica, MA, USA), which had a DIFFRACplus XRD Commender (Bruker AXS) software, as well as the Bragg–Brentano diffraction method, Θ—Θ, coupled in a vertical configuration with the following parameters: CuKα radiation, 2Θ region of 20–124, 2Θ step of 0.020, time of 8.7 s/step, rotation speed of 15 rot/min. Using the reference intensity ratio (RIR) method, a semi-quantitative evaluation of the identified phase concentrations was performed. The method was based upon scaling all the diffraction data to the diffraction levels of the standard reference materials. The results offer a qualitative indication of phase proportion in the alloy.

The Vickers microhardness of the alloys were determined at 25 °C using a micro-indenter attachment (Anton Paar MHT10, Anton Paar GmbH, Graz, Austria), which had an applied load of 2 N and a slope of 0.6 N/s.

## 3. Results

### 3.1. CCAs Structure Design Depending on Concentration

The redistribution of the solid solutions during the solidification process was studied by means of the MatCalc simulation program. The analysis tools obtained the equilibrium and Scheill–Gulliver diagrams, which provided information regarding the slow diffusion effect and the theoretical estimation of the solidification temperature.

The thermodynamic and kinetic criteria were calculated for the FeTaNbTiZr and FeMnNbTiZr alloy systems, in which the concentrations of each element were varied, to identify the most appropriate compositions for potential biocompatible applications.

**Figure 1:**
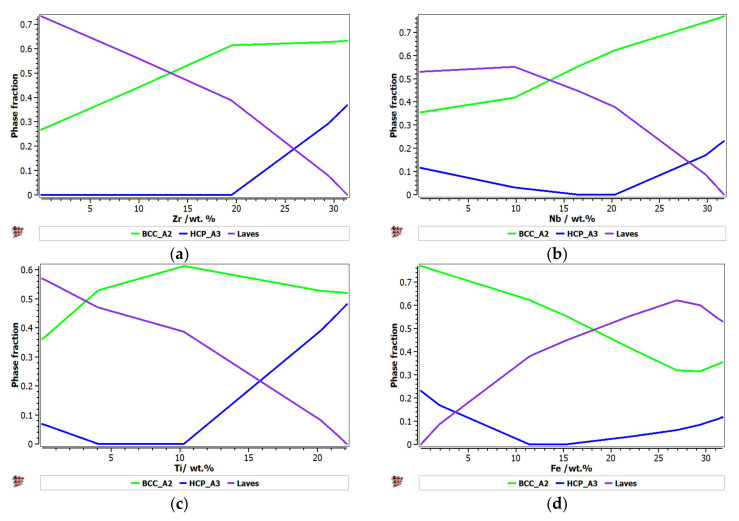

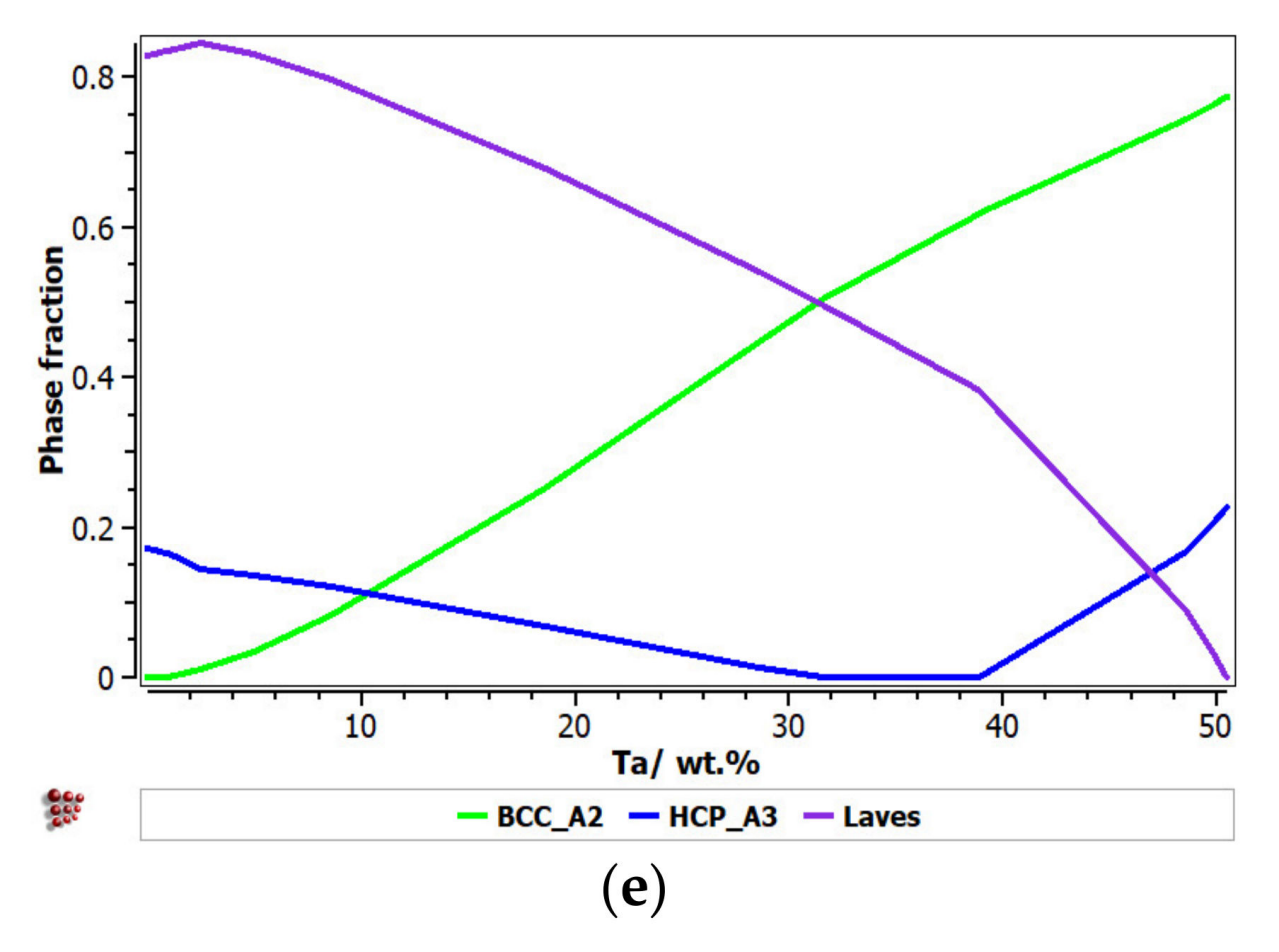
The proportions of phases that can be found in the FeTaNbTiZr alloy system at 200 °C, depending on the variation of (**a**) Zr; (**b**) Nb; (**c**) Ti; (**d**) Fe; (**e**) Ta.

Figure 1 presents the calculated phase distribution for the FeTaNbTiZr alloy system, which was dependent on the content of each constituent element. The step calculation simulation was performed by increasing the selected element concentration while maintaining proportional values for the rest of the elements.

It can be observed that the proportion of the BCC–A2 solid solution increased with the content of Zr, but the Laves intermetallic phase content decreased. At a percentage of 20%, Zr appeared to be A3 hexagonal, which is a brittle phase and specific to zirconium. Brittle phases are usually avoided in biocompatible alloys, so up to 20% of Zr is recommended to be used for alloy selection. The increase in the Nb content in the alloy had a similar result to Zr; with a proportion higher than 20%, and HCP–A3 phase starts to form. Up to 15% Nb, a significant proportion of the HCP–A3 phase can be observed, which was suppressed between 15 and 20% Nb. This concentration interval is recommended to be used for the intended application. Similar to Zr and Nb, Ti is important for stabilizing the BCC–A2 phase, up to 10%. When the titanium content exceeded 10%, the HCP–A3 phase started to form in the alloy structure. In Figure 1d, it can be observed that the presence of iron influenced the formation of phase structure by the inhibition of the BCC phase and the increase in the brittle HCP–A3 and Laves phases. Unlike the other elements, Ta had a constant influence on the formation of the BCC-A2 solid solution phase, up to a high concentration value of 50% (Figure 1e). The formation of the hardening HCP–A3 phase started at 40% Ta, which was a higher value compared to other elements. It was calculated the influence of brittle phases. As previously mentioned, the HCP–A3 phase was not desired in our configuration, and it was preferable, in this case, to have two-phase instead of three-phase structures. As a result, it was established that the optimal concentration interval for Ta was 30–40%.

**Figure 2:**
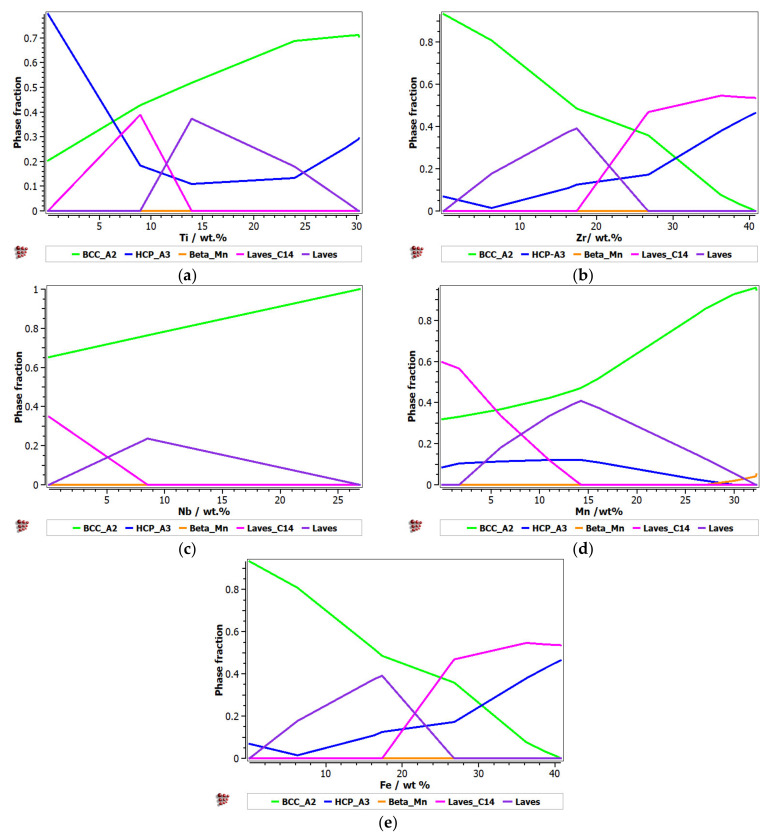
The proportions of phases that can be found in the FeMnNbTiZr alloy system at 200 °C, depending on the variations of (**a**) Ti; (**b**) Zr; (**c**) Nb; (**d**) Mn; (**e**) Fe.

For the second alloy system, FeMnNbTiZr, the influence of the alloying elements on the evolution of the constituent phases was analyzed (Figure 2). It can be observed that with an increase in the Ti percentage, there was a significant increase in the BCC–A2 solid solution phase and a decrease in the HCP–A3 hexagonal phase (Figure 2a). The Laves intermetallic phases had a maximum area of 0.4 M in the concentration interval of 8–15%Ti. A high percentage of 25–30%Ti is recommended for obtaining predominantly solid solutions in the alloy. The presence of Zr in the alloy at a percentage of up to 26% determined a decrease in the solid solution distribution and an increase in the content of Laves intermetallic phases (Figure 2b). A high concentration of Zr in CCA determined a decrease in the intermetallic compounds and an increase in the BCC–A2 and HCP–A3 solid solutions. When the Zr content was increased by more than 26%, the C14 Laves phase was reduced to a minimum. Nb has a high BCC–A2 phase stabilization capacity, higher than Zr and Ti, so at values over 27%, it can form exclusively solid solutions in the alloy (Figure 2c). In Figure 2d, it can be observed that the influence of Mn in the alloy system is significant. Up to 15% of Mn in the alloy formed a complex structure composed of the majority of the phases met in the system. At higher percentages, there was an accentuated stabilization of the main BCC–A2 phase, reaching a structure based mostly on the BCC–A2 solid solution at 32%. Similar to the FeTaNbTiZr alloy system, the influence of iron on the phase evolution in the FeMnNbTiZr system was significant, as it considerably decreased the BCC phase. An interval of up to 10 wt.% is recommended (Figure 2e).

### 3.2. Kinetic and Thermodynamic Criteria Calculation

**Figure 3:**
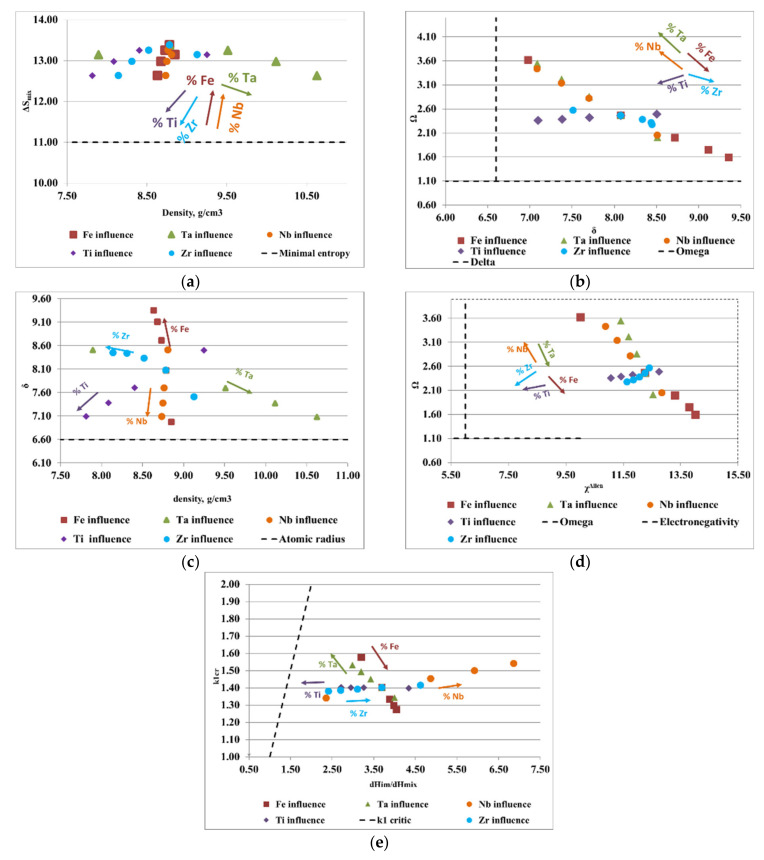
Graphical representation of the variations between (**a**) density, ρ, and the configurational entropy, ΔS_mix_; (**b**) the atomic radius difference, δ, and the derived parameter, Ω; (**c**) density, ρ, and the atomic radius difference, δ; (**d**,**e**) the ratio between the enthalpy of intermetallic compounds (ΔH_im_) and the mixing enthalpy (ΔH_mix_) with the k1_cr_ factor for the FeTaNbTiZr alloy system.

**Figure 4:**
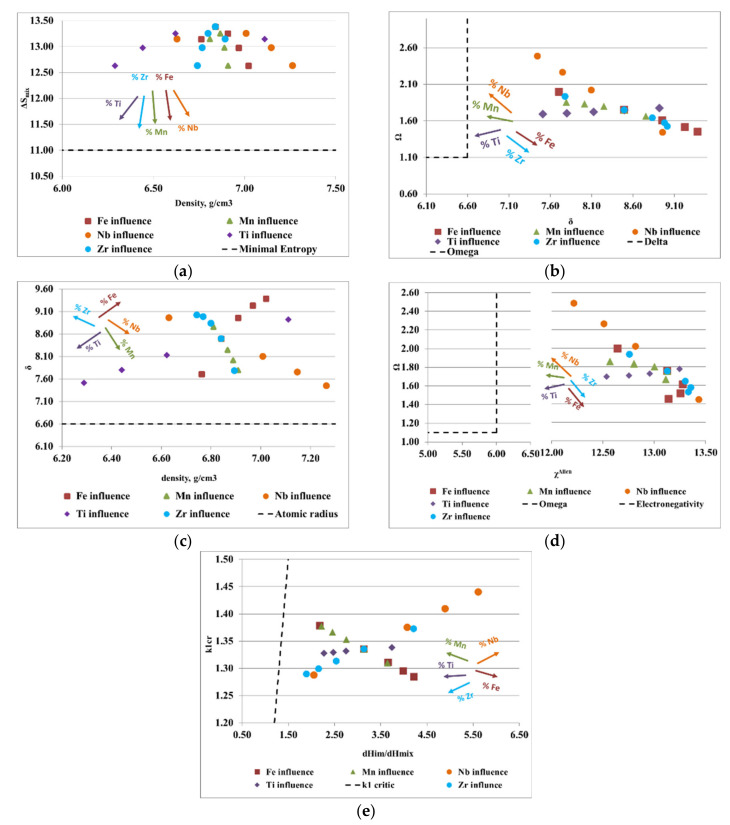
Graphical representation of the variations between (**a**) density, ρ, and the configurational entropy, ΔSmix; (**b**) the atomic radius difference, δ, and the derived parameter, Ω; (**c**) density, ρ, and the atomic radius difference, δ; (**d**) Allen electronegativity, χ^Allen^, and the derived parameter, Ω; (**e**) the ratio between the enthalpy of intermetallic compounds (ΔHim) and the mixing enthalpy (ΔHmix) with the k1_cr_ factor for the FeMnNbTiZr alloy system.

To design the optimal composition of complex concentrated alloys with reduced contents of critical raw materials and potential biocompatible properties, for both selected alloy systems (FeTaNbTiZr and FeMnNbTiZr), calculations of the thermodynamic criteria were performed, varying the proportions of each element. The influence of each constituent element proportion on the calculated parameters is shown in Figure 3 and Figure 4. The areas between the dotted lines represent the boundary intervals between which solid solutions can be formed, depending on the conditions of each parameter.

Figure 3a presents the ratio between the density of the alloy and the configurational entropy. It can be observed how the content of Ti, Zr, and Ta positively influenced the formation of solid solutions, while the increase in the content of Fe and Nb removed the alloy from the area considered optimal for the formation of solid solutions. Analyzing the variations of the derived parameter, Ω, with the atomic radius difference, δ (Figure 3b), it can be observed that an increase in the content of Ta, Nb, and Ti and a decrease in the content of Fe and Zr positively influenced the formation of solid solutions. Figure 3c illustrates the variations of the atomic radius difference, δ, with the density, ρ. Analyzing the trend of the chemical elements in the FeTaNbTiZr alloy system, it can be observed that increasing the content of Ta, Ti, and Nb, and decreasing the Fe and Zr content, positively influenced the formation of solid solution structures. Figure 3d presents the variations of the derived parameter Ω with Allen electronegativity, where it can be noticed that the elements Ti and Zr had a direct influence on the formation of solid solutions. On the other hand, maintaining low values of Fe and Ta had a positive influence, due to the positive characteristics they offered to the alloy; however, an increase in the concentrations of these elements can negatively influence the formation of solid solutions. Figure 3e shows the variations of the ratio between the enthalpy of the intermetallic compounds and the mixture enthalpy, considering the k1_cr_ factor, where it can be estimated that an increase in the Ti and Ta contents had a positive influence on solid solutions formation, while Zr, Fe, and Nb are not good solid solution formers.

Figure 4a shows the ratio between the alloy density and mixing entropy, where Ti, Zr, Mn, Fe, and Nb have a positive influence on the formation of solid solutions. Analyzing the variation of the derived parameter, Ω, with atomic radius difference, δ (Figure 4b), it can be observed that an increase in Mn, Nb, and Ti and a decrease in Zr and Fe stimulates the formation of solid solutions. The variations of the atomic radius difference, δ, with the density, ρ (Figure 4c), provide information regarding the elements that are good solid solution formers, such as Mn, Ti, and Nb. Regarding the diagram (Figure 4d), where the influence of the Allen electronegativity on the derived parameter is presented, Ω, it can be noted that Ti, Mn, and Nb were good solid solution formers. Moreover, the fluctuation of the ratio between the enthalpy of intermetallic compounds (ΔH_im_) and the mixing enthalpy (ΔH_mix_) with the k1_cr_ factor provides information regarding the elements such as Ti, Mn, and Nb that are desirable for the composition of complex concentrated alloys.

### 3.3. CCA Selection Using the CALPHAD Method and Kinetic and Thermodynamic Criteria

With the analysis of the CALPHAD modeling results for the FeTaNbTiZr alloy system, it was observed that a structure that was preponderantly based on solid solutions was obtained at high percentages of Ta [29], Nb, and Zr. Otherwise, the Ti had to be kept at relatively low percentages. By replacing the Ta with Mn in the FeMnNbTiZr alloy system, the most important influence on the formation of a structure composed of solid solutions was Zr [30].

The criteria calculation results provided information regarding the alloys’ structures, which were influenced by the proportion of constituent elements, which can have a positive or negative influence on the formation of solid solutions. For the FeTaNbTiZr alloy system, the Ta and Nb had a favorable influence on the evolution of the Ω and δ parameters, while the Ti reduced the value of density and improved the k1_cr_ critical factor. However, according to the European Commission list (Table 1), titanium is considered a critical metal, which implies that a sustainable alloy design has low Ti content, in terms of the FeTaNbTiZr alloy system. Although Ta increased the density of the alloy, it contributed substantially to the improvement of the selection criteria. Because Ta is also included in the critical raw material list (Table 1), a substitution with Mn was suggested, due to the similar solid solution formation behavior that both metals develop in combination with other elements, thus obtaining a new alloy system, FeMnNbTiZr.

The modeling results show a good capability of the selected systems to form structures based majorly on solid solution phases. It has been shown before that high percentages of Ta, Ti, Nb, Zr, and Mn favor the formation of BCC-type solid solution structures, while Fe has a negative influence on the formation of hard HCP–A3 and Laves phases. The results of the criteria simulations also show that Zr and Ta could have a negative impact on the final structure, as opposed to the CALPHADS modeling results. Suggestions made by the CALPHAD and criteria optimization were taken into account in the selection of the alloy compositions. However, considering the high cost of some elements (e.g., Ta and Nb) and their scarcity, the selection of the appropriate composition changed considerably. In this case, it was preferable to have higher contents of Fe and Mn, as well as lower contents of Ta, Nb, and Ti, which are considered critical metals. However, the simulation results show a favorable influence of the critical elements on the formation of solid solution structures. A compromise composition was selected in order to meet all the proposed requirements, after several preliminary trials. Thus, three new CCAs with low contents of critical raw materials and inexpensive obtaining processes were developed—FeTa_0.5_Nb_0.5_TiZr_0.5_, FeTa_0.5_Nb_0.5_Ti_1.5_Zr_0.5_, and FeMnNb_0.5_TiZr_0.5_. The Ti presence was still significant, even though it is an unwanted critical element, so a comparison was done with a lower Ti content for the second alloy.

The thermodynamic and kinetic criteria, calculated for the selected complex concentrated alloys, are presented in Table 2.

**Figure 5:**
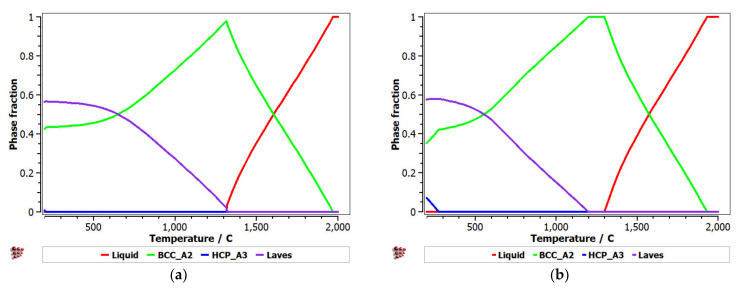
Equilibrium diagrams for the (**a**) FeTa_0.5_Nb_0.5_TiZr_0.5_ and (**b**) FeTa_0.5_Nb_0.5_Ti_1.5_Zr_0.5_ alloys.

**Figure 6:**
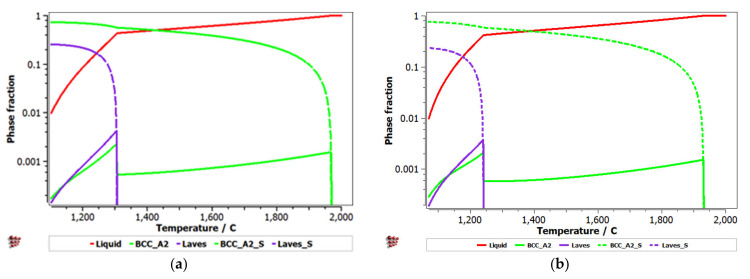
Scheill–Gulliver diagrams for the (**a**) FeTa_0.5_Nb_0.5_TiZr_0.5_ and (**b**) FeTa_0.5_Nb_0.5_Ti_1.5_Zr_0.5_ alloys.

Equilibrium and Scheil–Gulliver diagrams were calculated for the selected CCA compositions to determine the phase proportions identified in the alloy in specific solidification conditions. In the FeTaNbTiZr alloy system, it can be observed that the complex concentrated alloy with a high Ti content had a higher content of Laves intermetallic phases at room temperature (Figure 5). However, titanium made a considerable contribution to increasing the biocompatible properties of the alloy, so it was absolutely necessary to include it in the final composition. The Scheil–Gulliver diagrams (Figure 6) indicate high stability of the BCC–A2 phase, which was formed first during the solidification process. Thus, the Laves intermetallic phases mostly formed in the interdendritic area of the alloy.

**Figure 7:**
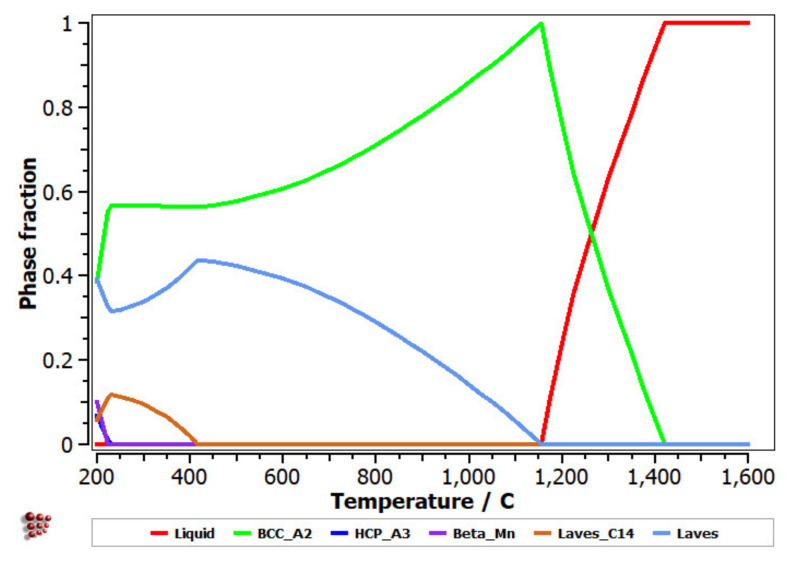
Equilibrium diagram of the FeMnNb_0.5_TiZr_0.5_ alloy.

The equilibrium diagram of the FeMnNb_0.5_TiZr_0.5_ (Figure 7) indicates the high stability of the solid solution, based on a BCC–A2 structure. At low temperatures, the Laves intermetallic phases had high contents, which determined a complex multiphase structure with low percentages of the HCP–A3 and Beta–Mn phases.

**Figure 8:**
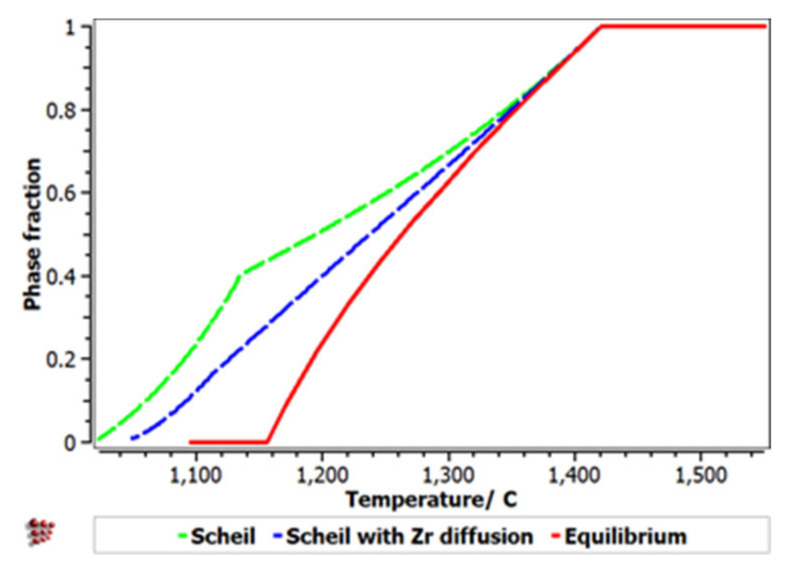
Scheil–Gulliver cooling diagram with Zr diffusion for the FeMnNb_0.5_TiZr_0.5_ alloy.

The Scheill–Gulliver solidification curve (Figure 8) indicates a high degree of subcooling. The model of the solidification process of the alloy and considering Zr diffusion and its impact on changing the structure in the solidification area, a decrease in the subcooling degree can be observed. 

**Figure 9:**
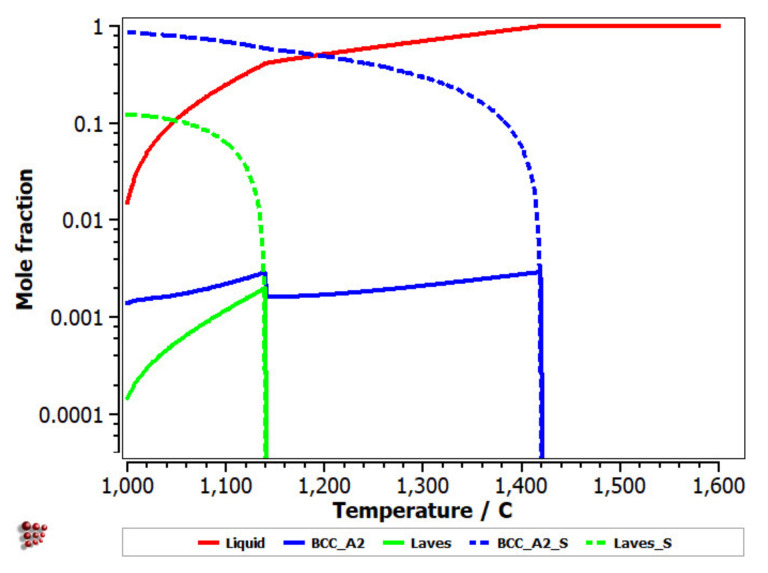
Scheill–Gulliver diagrams for FeMnNb_0.5_TiZr_0.5_.

With the analysis of the equilibrium diagram, it can be observed that the high stability of the BCC–A2 phase determined a primary solidification at a temperature of 1420 °C, according to the Scheil–Gulliver solidification diagram (Figure 9). The process was followed by the formation of the Laves intermetallic phase at a temperature difference lower than 200 °C.

### 3.4. Empirical Results

The alloys were examined in the as-cast state using chemical analysis, scanning electron microscopy (SEM), and X-ray diffraction (XRD).

The chemical and physical analysis results are presented in Table 3. The resulting alloy composition was found to be within 3% of the limits of the nominal values for the used elements [31].

**Figure 10:**
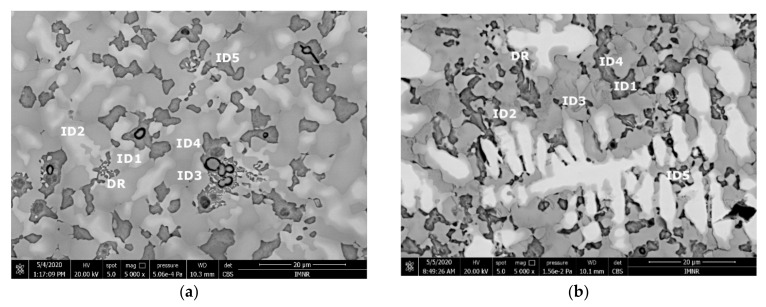
SEM-EDS image of the FeTa_0.5_Nb_0.5_Ti_1.5_Zr_0.5_ alloy in as-cast (**a**) and re-melted (**b**) states.

The SEM analyses of the as-cast and remelted FeTa_0.5_Nb_0.5_Ti_1.5_Zr_0.5_ alloy showed different types of dendritic structures with variable phase compositions from the center to the borders (Figure 10a,b). The remelted alloy presented larger and branched dendrites compared to the as-cast sample, which presented small and well-dispersed dendrite fragments. Six separate phases were distinguished in both structures. According to the EDS analysis, the dendrite area (DR) contained more Nb, Ti, and Ta (Figure 10) in the melted (Table 4) and re-melted (Table 5) FeTa_0.5_Nb_0.5_Ti_1.5_Zr_0.5_ alloys. The interdendritic area contained two phases–the phase corresponding to ID1 contained Ti, Fe, Zr, and small quantities of Ta and Nb. The phase corresponding to ID2 contained Ti, Nb, Fe, and small quantities of Ta and Zr. Additionally, in the interdendritic area, the presence of a eutectic with fine lamellar morphology in an as-cast sample and with fine punctiform distribution in the remelted state (ID3) was observed, which, according to the EDS analysis on the component corresponding to the ID3 point, contained high percentages of Ti, Fe, Zr, and less Ta and Nb. In the case of the component corresponding to ID4, a predominant Ti and Fe composition was distinguished; the rest of the elements were at lower values but still significant. A small phase that was identified in the interdendritic area is indicated by ID5, with different composition gradients for the two samples. The as-cast sample showed a predominant Ti and Fe composition, and the re-melted sample showed a predominant Ti, Nb, and Ta composition.

**Figure 11:**
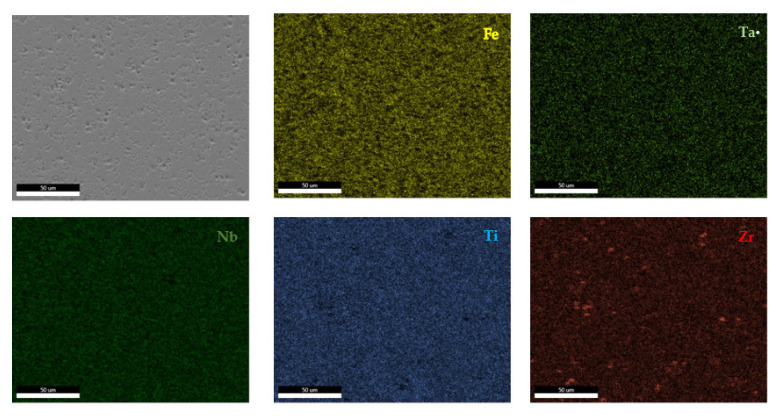
EDS mapping of the FeTa_0.5_Nb_0.5_Ti_1.5_Zr_0.5_ alloy.

**Figure 12:**
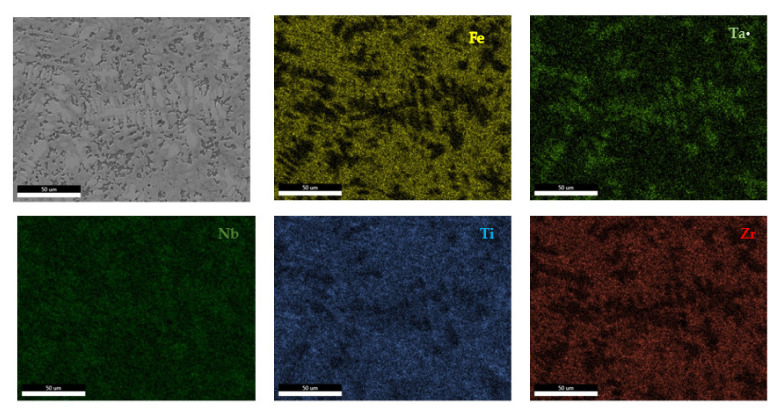
EDS mapping of the remelted FeTa_0.5_Nb_0.5_Ti_1.5_Zr_0.5_ alloy.

The EDS results (Figure 11 and Figure 12) show large differences in terms of dendrite size and element distribution. The as-cast alloy presented a homogenous structure with uniform element distributions, besides small size segregations for Zr. The re-melted sample presented well-defined dendrites with a high concentration of Ta, and less Nb and Ti. The interdendritic area was characterized by a major presence of Fe and Zr.

**Figure 13:**
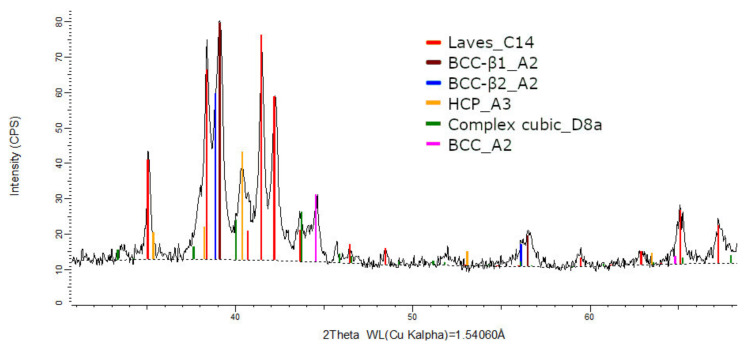
X-ray diffraction pattern for the FeTa_0.5_Nb_0.5_Ti_1.5_Zr_0.5_ alloy.

**Figure 14:**
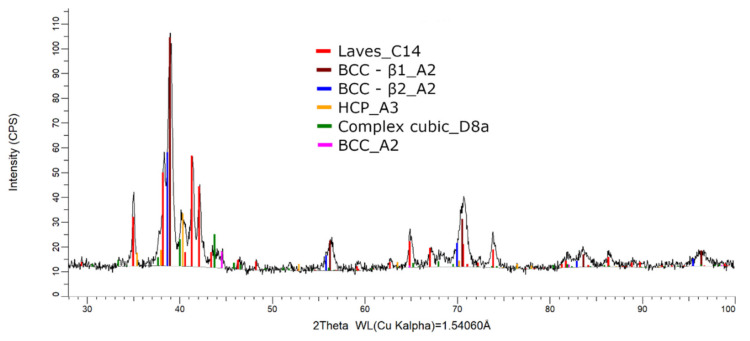
X-ray diffraction pattern for the remelted FeTa_0.5_Nb_0.5_Ti_1.5_Zr_0.5_ alloy.

The XRD phase analysis for the melted (Figure 13) and remelted (Figure 14) FeTa_0.5_Nb_0.5_Ti_1.5_Zr_0.5_ alloys revealed similar structure configurations, showing three different BCC–A2 type phases, a Laves phase, a complex cubic phase, and a hexagonal compact phase. Phase proportion calculations revealed high contents of Laves–C14 and BCC–β1–A2 phases, which changed between the two samples from 40 wt.% and 20 wt.% to 32 wt.% and 31 wt%, respectively. The other phases, BCC—β2–A2 (14 wt.%), HCP–A3 (13 wt.%), complex cubic–D8a (8 wt.%), and BCC–A2 (5 wt.%) had minor proportions. The high number of phases suggests a less stable structure that needs special heat treatment processing to reach the equilibrium configurations.

**Figure 15:**
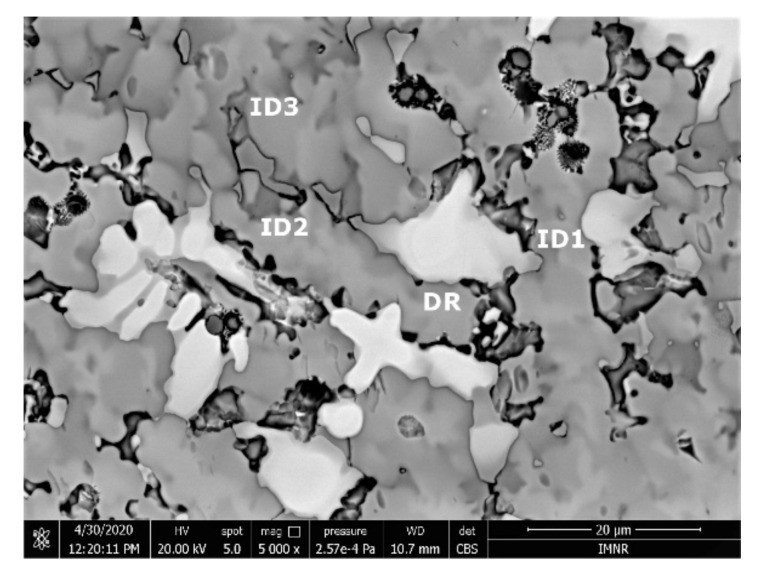
SEM-EDS images for the as-cast FeTa_0.5_Nb_0.5_TiZr_0.5_ alloy sample with the selected points for EDS analysis.

**Figure 16:**
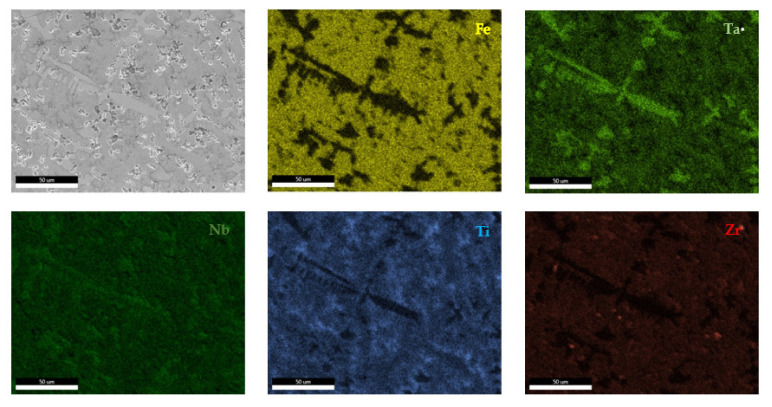
EDS mapping of FeTa_0.5_Nb_0.5_TiZr_0.5_ alloy.

The SEM analyses of the FeTa_0.5_Nb_0.5_TiZr_0.5_ alloy revealed a dendritic structure (Figure 15) with larger branches than for the FeTa_0.5_Nb_0.5_Ti_1.5_Zr_0.5_ alloy. According to the EDS analysis (Table 6 and Figure 16), the dendritic area (DR) contained mostly Ta, followed by Nb and smaller quantities of Ti and Fe. The interdendritic area (ID2) contained Ta, Fe, Ti, and smaller quantities of Zr and Nb. A smaller phase was distinguished in the interdendritic area, with a major proportion of Ti and less Ta, Nb, Fe, and Zr (ID3). Additionally, in the interdendritic area, the presence of another phase that contained mostly Ti, Fe, Zr, and smaller quantities of Ta and Nb (ID1) was observed.

**Figure 17:**
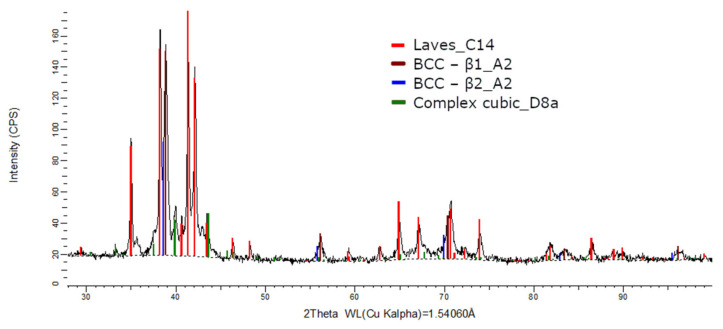
X-ray diffraction pattern for the FeTa_0.5_Nb_0.5_TiZr_0.5_ alloy.

Compared to the alloy with a higher Ti concentration, the FeTa_0.5_Nb_0.5_TiZr_0.5_ alloy showed a reduced phase composition in the XRD analysis report (Figure 17). Several structures, BCC–β1, BCC–β2, Laves phase, and the complex cubic phase, were detected. Due to the phase proportion calculations, it was observed that the laves–C14 phase (56 wt.%) was predominant, followed by BCC–β1–A2 (22 wt.%) phase, BCC–β2–A2 (12 wt.%), and complex cubic–D8a (10 wt.%). The XRD results for the FeTa_0.5_Nb_0.5_TiZr_0.5_ alloy show fewer phases than the alloy with a higher Ti content, but unstable BCC–A2 variations are still present.

**Figure 18:**
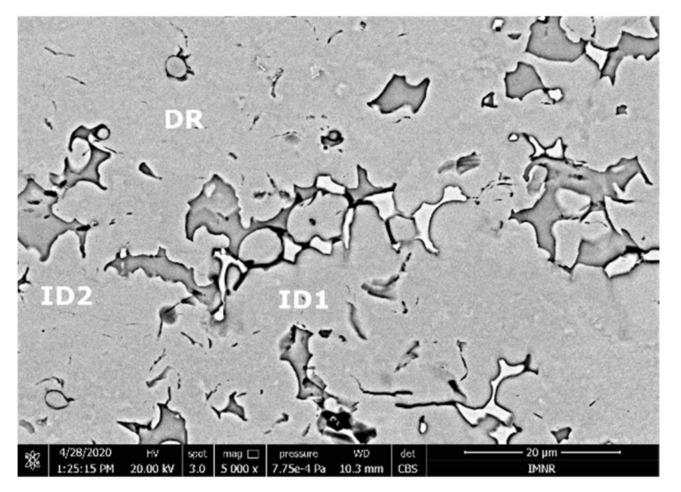
SEM image of the FeMnNb_0.5_TiZr_0.5_ alloy sample with the selected points for EDS analysis.

**Figure 19:**
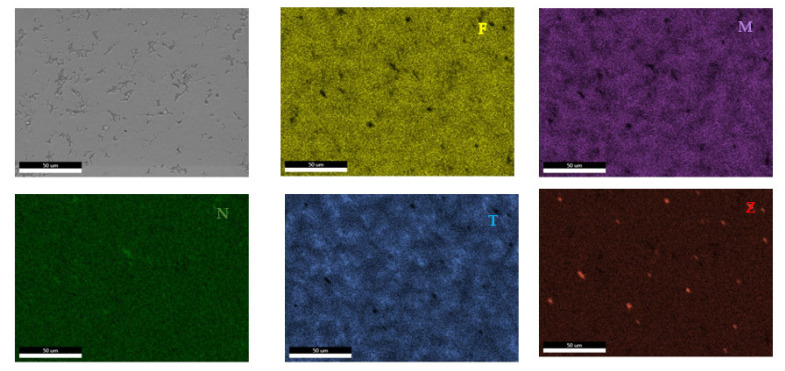
EDS mapping of the FeMnNb_0.5_TiZr_0.5_ alloy.

According to the SEM images (Figure 18), the FeMnNb_0.5_TiZr_0.5_ alloy sample had a large dendritic structure. The EDS analysis (Table 7 and Figure 19), shows that the dendritic area (DR) contained mostly Mn, Ti, and smaller quantities of Fe, Zr, and Nb. In the interdendritic area, the presence of two phases can be observed (ID1 and ID2). These phases had a high concentration of Ti, followed by Nb, Mn, Fe, and Zr.

**Figure 20:**
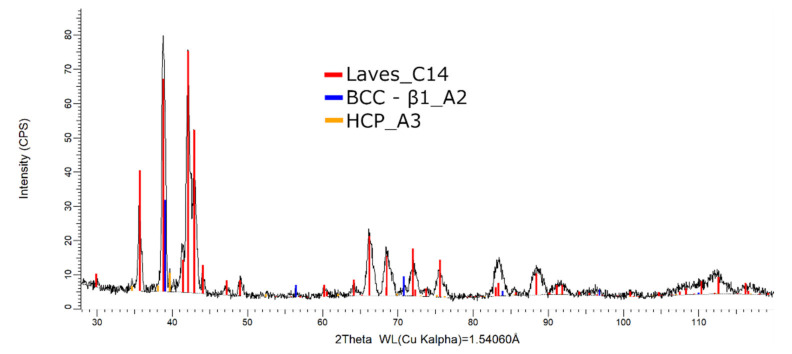
X-ray diffraction pattern for the FeMnNb_0.5_TiZr_0.5_ alloy.

The XRD phase analysis, presented in Figure 20, reveals several structures—the BCC–β1, Laves, and hexagonal compact phases. The Laves–C14 phases were present in a higher proportion (82 wt.%) than the BCC–β1–A2 (14 wt.%) and HCP–A3 (4 wt.%).

The samples were analyzed to determine the microhardness (Table 8) and it was observed that the FeTa0.5Nb0.5TiZr0.5 was the alloy with the highest microhardness (898.02 HV), followed closely by FeTa_0.5_Nb_0.5_Ti_1.5_Zr_0.5_ and FeMnNb_0.5_TiZr_0.5_. After the re-melting of the FeTa_0.5_Nb_0.5_Ti_1.5_Zr_0.5_ alloy, the microhardness decreased by approximately 13%.

The selection criteria applied to the FeTa_0.5_Nb_0.5_TiZr_0.5_, FeTa_0.5_Nb_0.5_Ti_1.5_Zr_0.5_, and FeMnNb_0.5_TiZr_0.5_ alloys showed good potential for the formation of mixed structures containing either solid solutions (SS) or intermetallic compounds (IM). The experimental findings match the criteria calculations for the FeTa_0.5_Nb_0.5_TiZr_0.5_ and FeTa_0.5_Nb_0.5_Ti_1.5_Zr_0.5_ alloys, where high proportions of SS and IM were distinguished. Nevertheless, for the FeMnNb_0.5_TiZr_0.5_ alloy, the experimental findings show much larger contents of intermetallic Laves-type phases than the criteria calculation results.

The CALPHAD method produced similar phase configurations with those obtained in the experimental results for the FeTa_0.5_Nb_0.5_TiZr_0.5_ and FeTa_0.5_Nb_0.5_Ti_1.5_Zr_0.5_ alloys, where BCC and Laves phases had similar proportions. The thermodynamic simulation for the FeMnNb_0.5_TiZr_0.5_ alloy showed a significantly lower proportion of the Laves phase in the experimental results.

The phases that were present in both representations for the FeTaNbTiZr system were BCC–A2, HCP–A3, and Laves. A complex D8_a_ phase was identified in the as-cast alloy sample and was not indicated by the CALPHAD simulation. The simulation of FeMnNbTiZr solidification behavior showed a larger number of phases (BCC–A2, HCP–A3, β–Mn, and Laves) than in the experimental results (Laves, BCC–A2 and HCP–A3). Overall, the results obtained in the experimental trials relate well to the preliminary alloy structural modeling results, and the differences encountered in the phase composition can be attributed to the nonequilibrium nature of the casting process.

## 4. Conclusions

This paper presents the selection process and characterization of complex concentrated alloys with reduced contents of critical raw materials. With the analysis of the properties of the main elements used for obtaining complex concentrated alloys with reduced contents of critical raw materials, two alloy systems are proposed—FeTaNbTiZr and FeMnNbTiZr. Considering the variations of the contained elements, the MatCalc simulation program was used to analyze the solid solutions’ redistribution during the solidification process. To design the optimal composition for both selected alloy systems, calculations of the kinetic and thermodynamic criteria were performed, varying the proportions of each element. For the FeTaNbTiZr alloy system, it was identified that high percentages of Ta, Nb, and Zr stimulate the formation of structures based on solid solutions. Ti has good biocompatible properties, but it is important it is kept at low percentages because it is included in the critical raw materials list. Besides reducing the Ti content, the substitution of Ta with Mn was also considered because of its high availability and known potential for the formation of solid solution structures, thus leading to the second selection for the alloy system—FeMnNbTiZr.

From the simulation results performed by CALPHAD modeling, the semi-empirical criteria calculations, and the critical characteristics of the used elements, several compositions were selected for further analysis—FeTa_0.5_Nb_0.5_Ti_1.5_Zr_0.5_, FeTa_0.5_Nb_0.5_TiZr_0.5_, and FeMnNb_0.5_TiZr_0.5_.

The selected alloys were prepared in an induction furnace for improved homogeneity and reduced processing stages. Significant structural changes were observed after the remelting process, shown for the FeTa_0.5_Nb_0.5_Ti_1.5_Zr_0.5_.

The empirical results provided by SEM-EDS and XRD analyses of the FeTa_0.5_Nb_0.5_Ti_1.5_Zr_0.5_ (melted and remelted), FeMnNb_0.5_TiZr_0.5_, and FeTa_0.5_Nb_0.5_TiZr_0.5_ alloys indicate a significant content in solid solutions as well as in intermetallic phases. The as-cast FeTa_0.5_Nb_0.5_Ti_1.5_Zr_0.5_ alloy presented a refined structure, containing a preponderant BCC–β1–A2 solid solution phase and characterized by different types of dendritic structures, with a variable phase composition from the center to the borders. The structure of the remelted alloy can be described by larger and branched dendrites, in comparison to the small and well-dispersed dendrite fragments of the melted composition. The differences between the melted and remelted FeTa_0.5_Nb_0.5_Ti_1.5_Zr_0.5_ alloys, in terms of dendrite size and element distribution, were observed in the EDS analysis.

The as-cast FeTa_0.5_Nb_0.5_Ti_1.5_Zr_0.5_ alloy showed a dendritic structure with uniform element distribution, besides small size segregations of Zr, while the remelted one presented well-defined dendrites. The as-cast FeTa_0.5_Nb_0.5_TiZr_0.5_ alloy sample, where the titanium content was reduced, had a small number of phases compared to the FeTa_0.5_Nb_0.5_Ti_1.5_Zr_0.5_ alloys (melted and remelted), and the dendritic structures have larger branches. The CCA composition based on manganese, FeMnNb_0.5_TiZr_0.5_, had a smaller number of phases and a large dendritic structure.

Considering the XRD results, it can be observed that the melted and remelted FeTa_0.5_Nb_0.5_Ti_1.5_Zr_0.5_ alloys showed three different BCC–A2-type phases—a Laves phase, a complex cubic phase, and a hexagonal compact phase, with similar phase proportions. With the decrease in Ti content, the number of phases decreased compared to the previous alloy, but unstable BCC-A2 variations were still present. The FeMnNb_0.5_TiZr_0.5_ alloy composition presented a structure based on BCC–β1–A2, Laves, and hexagonal compact phases.

By comparing the criteria and with the MatCalc simulation with the empirical results, it was observed that several variations of the BCC–A2 phase were present in the prepared samples, while more intermetallic phases were present in the simulation diagrams. However, the structural findings are similar in terms of phase constitution. BCC–A2, HCP–A3, and Laves appeared in both representations.

The studied alloys represent good options for further studies on the replacement and improvement of conventional alloys with biocompatibility properties.

## Figures and Tables

**Table 1:** Critical raw materials list [9].

Critical Raw Materials in 2020				
Antimony	Fluorspar	Magnesium	Scandium	Titanium
Baryte	Gallium	Natural graphite	Silicon	Strontium
Beryllium	Germanium	Natural rubber	Tantalum	
Bismuth	Hafnium	Niobium	Tungsten	
Borate	Heavy rare earth elements	Platinum group metals	Vanadium	
Cobalt	Light rare earth elements	Phosphate rock	Bauxite	
Coking Coal	Indium	Phosphorus	Lithium	

**Table 2:** Thermodynamic and kinetic criteria for the formation of optimal CCAs.

Alloy	∆S_mix_, J/mol•K	∆H_mix_, kJ/mol	δ, %	VEC, %	∆χ^Allen^, %	T_m_	Ω	∆H_im_	k1_cr_	ρ, g/cm^3^
FeTa_0.5_Nb_0.5_Ti_1.5_Zr_0.5_	12.42	−15.56	8.02	5.25	12.78	2201.78	1.76	−15.56	1.31	7.61
FeTa_0.5_Nb_0.5_TiZr_0.5_	12.89	−16.41	8.52	5.43	13.28	2239.01	1.76	−16.41	1.31	8.06
FeMnNb_0.5_TiZr_0.5_	12.97	−15.38	8.23	5.88	12.92	1927.40	1.63	−15.38	1.33	6.65

**Table 3:** The chemical composition and density of alloys.

Alloy	Composition Tip	wt.%						Density g/cm^3^
		Ti	Fe	Mn	Nb	Ta	Zr	
FeTa_0.5_Nb_0.5_Ti_1.5_Zr_0.5_	Analyses	24.17	19.15		15.55	27.56	13.57	7.61
	Nominal	23.14	18.02	-	14.97	29.17	14.7	
FeMnNb_0.5_TiZr_0.5_	Analyses	17.89	21.73	22.36	22.04		15.97	6.65
	Nominal	19.09	22.28	21.92	18.53	-	18.18	
FeTa_0.5_Nb_0.5_TiZr_0.5_	Analyses	18.83	21.21		15.27	29.74	14.95	8.06
	Nominal	16.73	19.51	-	16.22	31.62	15.92	
FeTa_0.5_Nb_0.5_Ti_1.5_Zr_0.5_ remelted	Analyses	19.2	18.42	-	15.8	25	12.4	7.61
	Nominal	22.16	20.28		16.39	27.52	13.65	7.61

**Table 4:** Phase compositions for the as-cast FeTa_0.5_Nb_0.5_Ti_1.5_Zr_0.5_ alloy.

Phase	Composition, wt.%				
	Zr	Nb	Ti	Ta	Fe
DR	4.17	30.30	43.39	14.38	7.76
ID1	14.16	4.01	45.47	2.35	34.01
ID2	6.88	15.23	51.36	5.53	20.99
ID3	11.98	7.18	45.62	3.74	31.48
ID4	9.76	14.75	41.87	9.03	24.59
ID5	11.59	9.03	41.48	5.66	32.24

**Table 5:** EDS compositions for the remelted FeTa_0.5_Nb_0.5_Ti_1.5_Zr_0.5_ alloy, in wt.%.

Phase	Composition, wt.%				
	Zr	Nb	Ti	Ta	Fe
DR	0.00	32.84	31.57	29.00	6.58
ID1	14.70	0.00	46.94	2.36	36.00
ID2	13.64	6.06	37.13	5.11	38.06
ID3	13.52	6.00	37.83	5.16	37.49
ID4	11.70	8.03	36.61	8.06	35.61
ID5	4.38	24.93	45.90	16.78	8.01

**Table 6:** EDS composition for the FeTa_0.5_Nb_0.5_TiZr_0.5_ alloy, in wt.%.

Phase	Composition, wt.%				
	Zr	Nb	Ti	Ta	Fe
DR	-	28.95	21.93	41.02	8.11
ID1	17.26	4.05	40.96	6.31	31.42
ID2	10.35	7.18	27.13	14.73	40.61
ID3	4.84	13.16	60.68	8.83	12.49

**Table 7:** EDS composition for the FeTa_0.5_Nb_0.5_TiZr_0.5_ alloy, in wt.%.

Phase	Composition, wt.%				
	Zr	Nb	Ti	Ta	Fe
DR	7.33	16.20	30.65	32.41	13.41
ID1	6.82	12.70	38.86	25.72	12.70
ID2	7.00	13.26	39.33	20.78	17.21

**Table 8:** Vickers microhardness for the obtained alloys.

Alloy	Vickers Microhardness, HV
FeTa_0.5_Nb_0.5_Ti_1.5_Zr_0.5_	802.8
re-melted FeTa_0.5_Nb_0.5_Ti_1.5_Zr_0.5_	699.0
FeTa_0.5_Nb_0.5_TiZr_0.5_	898.2
FeMnNb_0.5_TiZr_0.5_	802.9

## Data Availability

Not applicable.

## References

[1] Constantinescu D., Cârlan B.A. A short history of the iron and steel industry in Central Europe during the Roman Iron Age. Proceedings of the 25th International Conference on Metallurgy and Materials Metal 2016.

[2] Tylecote R.F. (1992). A History of Metallurgy.

[3] Santos G.A. (2017). The importance of metallic materials as biomaterials. J. Tissue Eng. Regen. Med..

[4] Wintermantel E., Mayer J., Ruffieux K., Bruinink A., Eckert K.L. (1999). Biomaterials, human tolerance and integration. Chirurg.

[5] Chen Q., Thouas G.A. (2015). Metallic implant biomaterials. Mater. Sci. Eng. R Rep..

[6] Silver F.H., Christiansen D.L. (1999). Biomaterials Science and Biocompatibility.

[7] Love B. (2017). Metallic Biomaterials.

[8] Gunnarsdóttir I., Davidsdottir B., Worrell E., Sigurgeirsdóttir S. (2021). Sustainable energy development: History of the concept and emerging themes. Renew. Sustain. Energy Rev..

[9] European Commission Report. Critical Raw Materials Resilience: Charting a Path towards Greater Security and Sustainability. Communication from the Commission to the European Parliament COM (2020) 474 Final, Brussels. https://www.europeansources.info/record/critical-raw-materials-resilience-charting-a-path-towards-greater-security-and-sustainability/.

[10] Hofmann M., Hofmann H., Hagelüken C., Hool A. (2018). Critical raw materials: A perspective from the materials science community. Sustain. Mater. Technol..

[11] Cantor B., Chang I.T.H., Knight P., Vincent A.J.B. (2004). Microstructural development in equiatomic multicomponent alloys. Mater. Sci. Eng..

[12] Gorsse S., Couzinié J.P., Miracle D.B. (2018). From high-entropy alloys to complex concentrated alloys. Comptes Rendus Phys..

[13] Gorsse S., Miracle D.B., Senkov O.N. (2017). Mapping the world of complex concentrated alloys. Acta Mater..

[14] Mukherjee S. (2020). Complex concentrated alloys (CCAs)—Current understanding and future opportunities. Metals.

[15] Zhang Y. (2008). Solid-solution phase formation rules for multi-component alloys. Adv. Eng. Mater..

[16] Manzoni A.M., Glatzel U. (2019). New multiphase compositionally complex alloys driven by the high entropy alloy approach. Mater. Charact..

[17] Syrovatka M. (2020). On sustainability interpretations of the ecological footprint. Ecol. Econom..

[18] Agren J. (2020). CALPHAD—An approach to predict microstructural stability. Mater. Sci. Eng..

[19] Zhang C., Zhang F., Chen S., Cao W. (2012). Computational Thermodynamics Aided High-Entropy Alloy Design. JOM.

[20] Senkov O.N., Miller J.D., Miracle D.B., Woodward C. (2015). Accelerated exploration of multi-principal element alloys with solid solution phases. Nat. Commun..

[21] Chen H., Mao H., Chen Q. (2018). Database development and Calphad calculations for high entropy alloys: Challenges, strategies, and tips. Mater. Chem. Phys..

[22] Lederer Y., Toher C., Vecchio K.S., Curtarolo S. (2018). The search for high entropy alloys: A high–throughput ab–initio approach. Acta Mater..

[23] Troparevsky M.C., Morris J.R., Kent P.R., Lupini A.R., Stocks G.M. (2018). Criteria for predicting the formation of single-phase high-entropy alloys. Phys. Rev..

[24] Takeuchi A., Inoue A. (2000). Calculations of Mixing Enthalpy and Mismatch Entropy for Ternary Amorphous Alloys. Mater. Trans. JIM.

[25] Yang X., Zhang Y. (2012). Prediction of high-entropy stabilized solid-solution in multi-component alloys. Mater. Chem. Phys..

[26] Poletti M.G., Battezzati L. (2014). Electronic and thermodynamic criteria for the occurrence of high entropy alloys in metallic systems. Acta Mater..

[27] Guo S. (2011). Effect of valence electron concentration on stability of fcc or bcc phase in high entropy alloys. J. Appl. Phys..

[28] Singh A.K., Kumar N., Dwivedi A., Subramaniam A. (2014). A geometrical parameter for the formation of disordered solid solutions in multi-component alloys. Intermetallics.

[29] Cardonne S.M., Kumar P., Michaluk C.A., Schwartz H.D. (1995). Tantalum and its Alloys. Int. J. Refract. Hard Met..

[30] Ferro P., Bonollo F. (2019). Materials selection in a critical raw materials perspective. Mater. Des..

[31] Cârlan Șerban B.A. (2020). Theoretical and Experimental Researches Regarding the Obtaining of New High Entropy Alloy with Biocompatible Properties. Ph.D. Thesis.

